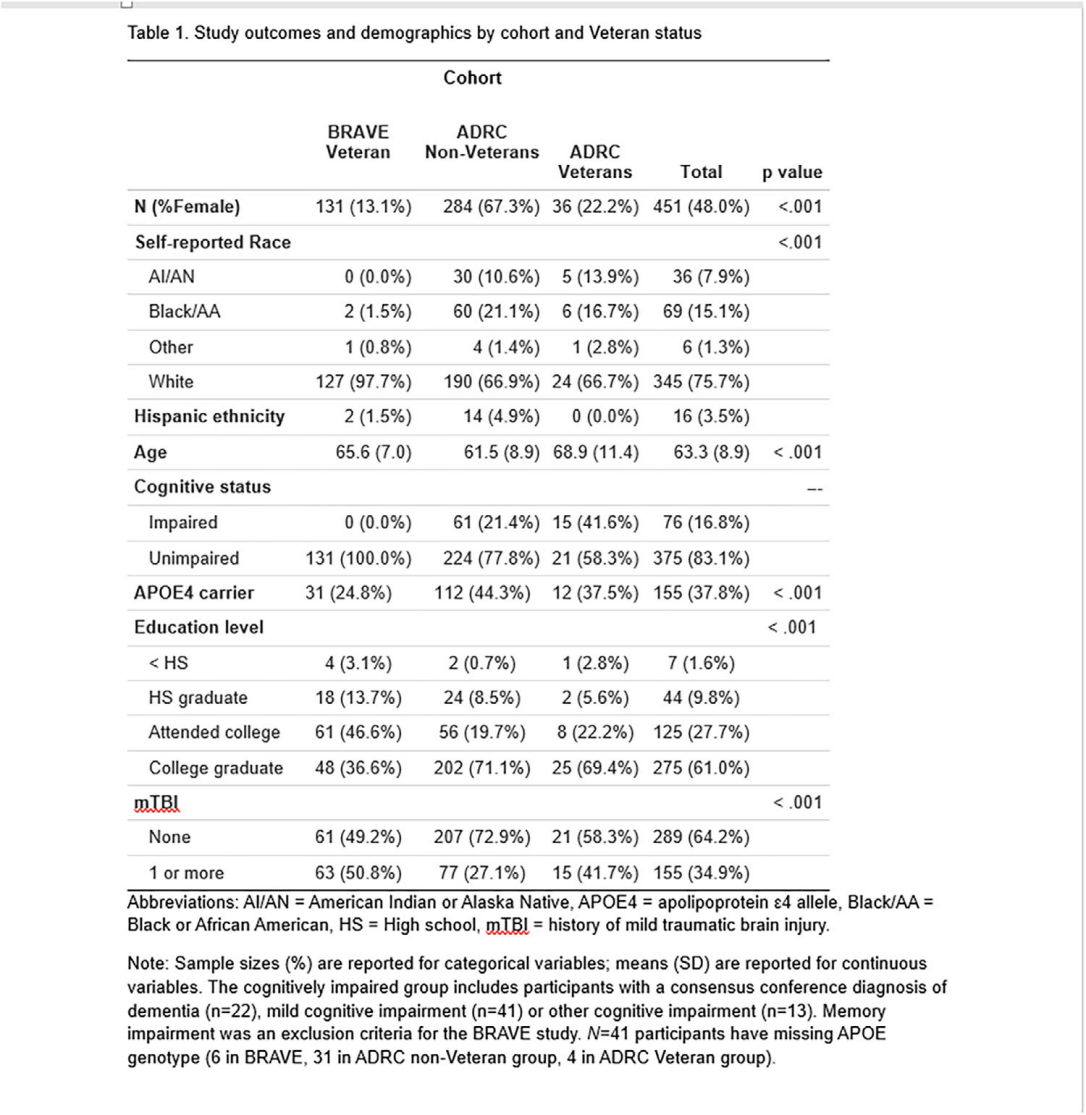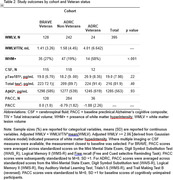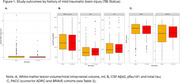# Association between remote Traumatic Brain Injury and AD pathology, vascular injury, and cognitive decline among Veterans and non‐Veterans

**DOI:** 10.1002/alz.091387

**Published:** 2025-01-03

**Authors:** Carol A. Van Hulle, Hanna Zylstra, Tobey J. Betthauser, Lianlian Du, Aleshia Cole, Katherine Cronin, Leonardo A. Rivera‐Rivera, Carey E. Gleason, Sterling C. Johnson, Henrik Zetterberg, Cynthia M. Carlsson

**Affiliations:** ^1^ Wisconsin Alzheimer’s Disease Research Center, University of Wisconsin School of Medicine & Public Health, Madison, WI USA; ^2^ Division of Geriatrics, Department of Medicine, University of Wisconsin School of Medicine and Public Health, Madison, WI USA; ^3^ School of Medicine and Public Health, University of Wisconsin‐Madison, Madison, WI USA; ^4^ Department of Medicine, Geriatrics Division, School of Medicine and Public Health (SMPH), University of Wisconsin‐Madison, Madison, WI USA; ^5^ Department of Medical Physics, University of Wisconsin‐Madison, Madison, WI USA; ^6^ Wisconsin Alzheimer’s Disease Research Center, School of Medicine and Public Health, University of Wisconsin‐Madison, Madison, WI USA; ^7^ Wisconsin Alzheimer’s Institute, University of Wisconsin‐Madison School of Medicine and Public Health, Madison, WI USA; ^8^ Wisconsin Alzheimer’s Disease Research Center, University of Wisconsin School of Medicine and Public Health, Madison, WI USA; ^9^ Geriatric Research, Education and Clinical Center (GRECC), William S. Middleton Memorial Veterans Hospital, Madison, WI USA; ^10^ University of Wisconsin School of Medicine and Public Health, Madison, WI USA; ^11^ Wisconsin Alzheimer’s Disease Research Center, Madison, WI USA; ^12^ Department of Medicine, University of Wisconsin‐Madison School of Medicine and Public Health, Madison, WI USA; ^13^ University of Wisconsin‐Madison School of Medicine and Public Health, Madison, WI USA; ^14^ Wisconsin Alzheimer’s Institute, University of Wisconsin School of Medicine and Public Health, Madison, WI USA; ^15^ Hong Kong Center for Neurodegenerative Diseases, Hong Kong China; ^16^ Sahlgrenska University Hospital, Gothenburg Sweden; ^17^ Institute of Neuroscience and Physiology, Sahlgrenska Academy at the University of Gothenburg, Mölndal, Gothenburg Sweden; ^18^ Dementia Research Centre, Department of Neurodegenerative Disease, UCL Queen Square Institute of Neurology, University College London, London, United Kingdom, London United Kingdom; ^19^ Wisconsin Alzheimer’s Disease Research Center, University of Wisconsin School of Medicine and Public Health, Madison, WI USA; ^20^ Department of Psychiatry and Neurochemistry, Institute of Neuroscience and Physiology, The Sahlgrenska Academy, University of Gothenburg, Mölndal, Gothenburg Sweden; ^21^ Wisconsin Alzheimer’s Institute, University of Wisconsin, Madison, WI, USA, Madison, WI USA; ^22^ VA Geriatric Research, Education and Clinical Center (GRECC), William S. Middleton Memorial Veterans Hospital, Madison, WI USA

## Abstract

**Background:**

Each year, millions of Americans experience mild traumatic brain injury (mTBI). Current research on the long‐term effects of mTBI vary considerably. Several mechanisms linking mTBI to dementia have been proposed including amyloid plaque formation and cerebrovascular injury following mTBI. Veterans have higher rates of mTBI than non‐Veterans. As efforts to increase Veteran enrollment in clinical trials and studies related to Alzheimer disease expand, it is critical to understand the potential impact of mTBI on cognitive ability and factors related to risk for AD. This study leveraged two cohorts to examine the association between history of mTBI and white matter hyperintensity lesion volume (WMLV), CSF pTau181, total tau, and Aβ42, and cognitive ability.

**Method:**

The sample comprises, *N* = 131 VA eligible Veterans without memory impairment enrolled in the Brain Amyloid and Vascular Effects of Eicosapentaenoic Acid (BRAVE) clinical trial (NCT02719327) and *N* = 320 participants in the Wisconsin ADRC clinical core, including n = 36 identified as Veterans. BRAVE participants reported if they had experienced symptoms of mTBI (e.g. loss of consciousness, dizziness, headache). ADRC participants indicated if they had a recent or remote mTBI; participants with a recent mTBI were excluded (n = 6).

CSF samples were assayed using elecsys® beta‐Amyloid(1_42) CSF II, phopho‐Tau (181P), and Total tau assays (Roche Diagnostics International, Switzerland) and log‐transfomed prior to analyses. WMHLV adjusted for TIV was binarized to WMH presence or absence. Separate Preclinical Alzheimer’s Cognitive Composite scores were created for BRAVE and were analyzed separately. Regression models included gender, age at study visit, clinical status and education level (PACC only) as covariates.

**Result:**

Demographics and study outcomes stratified by cohort and Veteran status are shown in Table 1 and 2. Veterans had higher rates of mTBI than non‐Veterans. History of mild TBI status was unrelated to study outcomes (see Figure 1; WMH presence: p = .06; CSF biomarkers: *p*s = .50 to .88; BRAVE‐PACC: *p* = .15, ADRC‐PACC, *p* = .48).

**Conclusion:**

History of mild TBI had no long‐term impact on cerebrovascular dysfunction, AD biomarkers or baseline cognitive performance. Further analyses will explore severity of TBI (with or without loss of consciousness) on study outcomes as well asexamine the impact of mTBIon change in cognitive performance and biomarkers of neuroinflammation.